# Variation in Growth, Wood Density, and Stem Taper Along the Stem in Self-Thinning Stands of *Sassafras tzumu*

**DOI:** 10.3389/fpls.2022.853968

**Published:** 2022-06-03

**Authors:** Songfeng Diao, Honggang Sun, David I. Forrester, Alvaro A. V. Soares, Thiago P. Protásio, Jingmin Jiang

**Affiliations:** ^1^Research Institute of Non-timber Forestry, Chinese Academy of Forestry/Key Laboratory of Non-timber Forest Germplasm Enhancement and Utilization of National Forestry and Grassland Administration, Zhengzhou, China; ^2^Research Institute of Subtropical Forestry, Chinese Academy of Forestry, Hangzhou, China; ^3^Swiss Federal Institute for Forest, Snow and Landscape Research, Birmensdorf, Switzerland; ^4^Department of Biology, Federal University of Uberlândia-UFU, Monte Carmelo, Brazil; ^5^Department of Wood Science and Technology, Rural Federal University of the Amazonia-UFRA, Parauapebas, Brazil

**Keywords:** Chinese sassafras, ring growth, stand density, stem form, tree ring wood density

## Abstract

Silvicultural practices greatly improve the economic value of wood products from forests. Stem dimensions, wood density, and stem form are closely linked to end-product performance. This research aimed to examine the effects of stand density and stem height on variables that reflect ring growth and wood properties of *Sassafras tzumu* stands during the self-thinning phase. Between the ages of 10 and 40 years, the number of stems per hectare has declined from 1,068 to 964 due to density-dependent mortality. As the relative stand density decreased, there were significant reductions in the average tree ring width (5.07–3.51 mm) and increases in latewood proportions (49.88–53.49%) and the density of the annual growth ring (165.60–708.58 kg/m^3^). Therefore, ring density, earlywood density, and latewood density increased with decreasing relative stand density after self-thinning occurred. Ring width, earlywood width, and latewood width significantly increased from the base to the apex of the stem. Stand density and stem height had additive effects on *S. tzumu* wood properties during the self-thinning phase. A shift in the growth allocation along the longitudinal stem in response to self-thinning resulted in decreasing radial growth, increasing wood density, and improved stem form. In summary, we found a significant influence of stand density on tree ring growth, wood quality, and stem form of *S. tzumu* trees during the self-thinning phase.

## Introduction

Silvicultural practices that affect tree growth may cause changes in the economic value of the raw material. Of the different intensive management treatments, stand density management is of critical importance to end-product properties (Moore et al., 2012; Zeller et al., 2017; Stankova and Diéguez-Aranda, 2020). For example, reducing the number of rows in a plantation while retaining a commercial stand density gives rise to greater financial return and lesser market risk associated with long rotation plantation forestry (Cassidy et al., 2013). Traditionally, while plantations with larger initial spacing are widely used to reduce the number of thinning treatments, shorten the rotation age, lower establishment costs, and accelerate the growth of individual trees, it has been recognized that this has negative effects, including greater stem taper and branch sizes. Concerns have been expressed regarding the effects of stand density on tree growth and wood quality during stand development (Moore et al., 2012; Dobner et al., 2018). On the one hand, the contradictory findings from numerous studies based on commercial tree species indicate the impacts of stand density on ring width, wood density, and stem form (Tong and Zhang, 2005; Lee and Choi, 2019).

On the other hand, relative stand density effects can be clearly detected in self-thinning stands as tree–tree competition results in density-dependent mortality (Sun et al., 2018). Studying with this type of stand can provide us the effects of stand density on tree growth and these effects would likely extend to the wood properties (Moore et al., 2012; Zhang et al., 2017). While several studies with *Tsuga heterophylla*, *Picea glauca*, and *Picea engelmannii* have examined tree growth and wood quality responses to competition, these studies focused on a specific tree height (e.g., 1.30 or 1.37 m) (Watson et al., 2003; Billah and Goldblum, 2019). Given that measurements obtained from a specific tree height provide limited information about the pattern of growth allocation and wood quality from the stem base toward the stem apex (Bevilacqua et al., 2005; Auty et al., 2014), it is important to sample from multiple heights to quantify stem quality and economic value. Therefore, deploying a stem analysis method to evaluate growth and wood properties along the longitudinal stem direction during stand self-thinning trajectory would provide a much more informed perspective of such patterns. In addition, deployment of a relative stand density metric rather than absolute stand density values would have been advantageous when inferring wood quality changes during the self-thinning phase.

*Sassafras tzumu* is one of the most important broad-leaved deciduous tree species in southern China (Li et al., 2019; Sun et al., 2020). It is a highly valued timber species with dark brown-red heartwood, which is often fragrant (Zhang et al., 2020). The wood has a medium density with considerable mechanical strength and high natural durability. Therefore, *S. tzumu* wood is suitable for carpentry and shipbuilding. Due to the importance of this species for the Chinese forest sector, the wood properties of this species, and how they change with stand density during the self-thinning phase, are of great interest. However, stand productivity of *S. tzumu* plantations is modified by self-thinning, age, site, and planting density (Sun et al., 2018), but very little is known about how growth, wood quality, and stem taper change along the stem (with increasing height from the base to apex), especially in self-thinning stands for the *S. tzumu.*

Therefore, this study aimed to examine the effects of stand density and longitudinal stem height on variables that reflect ring growth and wood properties of *S. tzumu* stands during the self-thinning phase. The aims of this study were to (i) investigate the influence of relative stand density and longitudinal stem position on diameter increment, wood density, and stem form; (ii) assess growth allocation along the stem from the stem base toward the stem apex; and (iii) detect whether there are interactive effects of stand density and longitudinal stem position on wood properties within each growth ring.

## Materials and Methods

### Study Site and Layout

**FIGURE 1:**
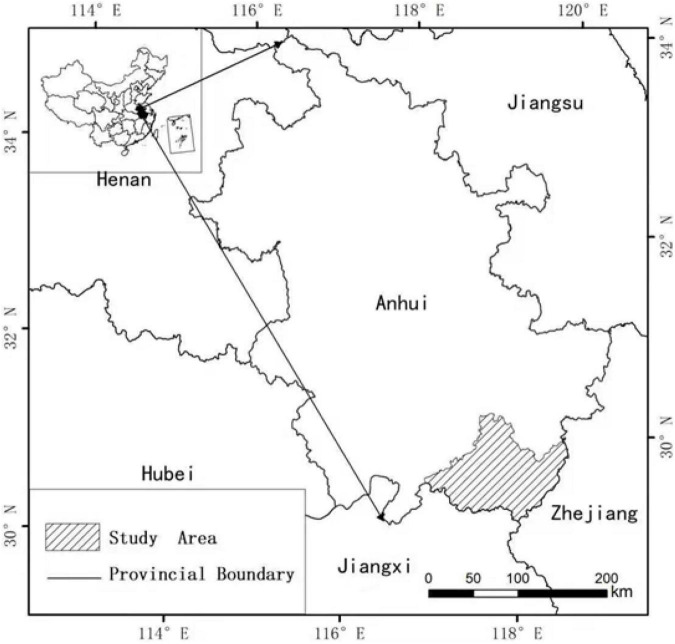
The location of the study area in China.

The study was conducted in the Huangshan Forestry Teaching Farm (latitude 30°10′N, longitude 118°11′E, elevation 674 m above sea level), in Mt. Huangshan, Anhui Province, China (Figure 1). The climate belongs to the subtropical monsoonal climatic zone and is characterized by long, warm summers and short, cool winters, with an average, maximum, and minimum annual temperature of 26.8, 31.4, and 22.7°C, respectively. Monthly mean, maximum, and minimum precipitation are 2,395, 1,496, and 2,956 mm, respectively. The average length of the growing season is 240 frost-free days. The study area has undulating topography and predominately yellow earth and stony till overlying bedrock. The soil is shallow, with exposed large boulders on ridge tops, although it is deeper in lower-lying areas. The high-quality site was located at the lower slope and had a mean annual increment of 13.1 m^3^/ha/year at the age of 20 years. The intermediate quality site was located at middle slope and had a mean annual increment of 11.7 m^3^/ha/year at the age of 20 years. The low-quality site was located at upper slope and had a mean annual increment of 8.5 m^3^/ha/year at the age of 20 years.

Six *S. tzumu* plots on different sites were established in 1977 at a planting spacing of 2.0 m × 3.0 m (1,667 stems/ha). The plot size was 20 m × 30 m (ten rows with ten trees) with a buffer zone consisting of 3 rows at the same spacing. This initial spacing was chosen because it is the most used planting spacing for the establishment of *S. tzumu* plantations.

### Measurements and Calculations

The trees in all the plots were numbered and measurements were conducted biannually, after the total tree height reached 1.30 m, from 1978 to 2018. The total tree height (m), and diameter at breast height (DBH, cm) were measured for all trees and the stem volume (*V*, m^3^) was estimated using the formula *V* = 5.0479055 × 10^–5^ × (DBH)^1.9085054^ × (TH)^0.99076507^ (Liu et al., 2001).

Relative stand density was calculated by living stem number per hectare divided by the maximum number of trees of a defined reference size (Pretzsch et al., 2017).

Summary statistics for the plots are presented in Table 1.

**TABLE 1:** Summary of the mean (±SE) of the variables investigated in the even-aged pure *S. tzumu* stands.

Stand age (years)	Stand density (stems ha^–1^)	Relative stand density	Tree DBH (cm)	Tree height (m)	Tree volume (m^3^)
10	1,068261	1.12	13.953.34	14.841.71	0.120.04
20	1,021200	1.07	15.192.51	15.114.02	0.150.03
30	988181	1.03	22.665.33	15.282.36	0.340.16
40	964114	1.01	24.997.29	16.973.52	0.410.08

Stand mortality rate was calculated for every other year from 1978 to 2018 as the percentage of dead trees in relation to the total of seedlings planted. The *S. tzumu* plantations began self-thinning in 1988, quantified as when the mortality rate exceeded 10% (Sun et al., 2011).

For the stem analyses, three individuals per plot of the average dominance class fell every 10 years in winter from 1988 to 2018. The trees were randomly selected from the buffer zones. The selection criteria for the sample trees were that they had a straight main stem with no forking or broken top and no obvious damage or wounds along the longitudinal stem direction. The main stem of the tree was divided into five sections in relation to total height (TH), at the following heights: stem base (0.15 m, stump height), breast height (1.30 m), 1/4 TH, 1/2 TH, and 3/4 TH.

A 5.0-cm-thick disk was removed from the base of each section to conduct detailed stem analyses on each subject tree in the laboratory. The disks were stored in air-dried rooms with 12% equilibrium moisture content for up to 8 months before being prepared for tree-ring measurements. Disk preparation involved sanding the bottom surface of each disk with sandpaper with the 40-grain size of decreasing coarseness and avoiding the oxidation of the wood surface until the annual rings were clearly recognized. On each stem disk, the following variables were determined: ring width (RW, mm), earlywood width (EWW, mm), latewood width (LWW, mm), latewood proportion (LWP, %), ring density (RD, kg/m^3^), earlywood density (EWD, kg/m^3^), and latewood density (LWD, kg/m^3^). These variables were measured using a high-frequency densitometry measurement LignoStation™ system (Rinntech,™ Heidelberg, Germany) with 1/1,000 mm along four radii taken 90° from each other, with the initial radius selected at random (Vannoppen et al., 2019). Latewood proportion (LWP) was calculated for each ring by dividing LWW by RW × 100.

Stem taper (ST, cm/m), butt taper (BT, cm/m), and the ratio of the diameter at different sampling heights to the diameter at breast height (*d*_*i*_/DBH, *i* = stem base, 1.30 m TH, 1/4 TH, 1/2 TH, and 3/4 TH) were used to assess stem taper (Mäkinen and Isomäki, 2004; Castle et al., 2018). ST was measured as the diameter difference between stem base and the apex 6.0 cm stem diameter along merchantable timber. Given that the butt log is generally the most valuable part of a tree, BT was determined for the first 2.5 m log from the butt for each stem.

Tree-ring indices were used to discriminate between juvenile and mature wood based on the following five steps: (1) the age-related ring growth trends were quantified (Pritzkow et al., 2014); (2) the heteroscedasticity of tree-ring chronology was stabilized by power transformation (Cook and Peters, 1997); (3) the Hugershoff equation was used to remove age-related ring growth trends (Cook et al., 1990); (4) age-related ring chronology was induced by their average values; and (5) a discontinuous linear regression equation was used to define the juvenile wood and the mature wood in the cross-section disks along with the different stem height (Yang et al., 1986).

### Statistical Analysis

We used a generalized linear mixed-effect model to analyze the relationships between relative stand density and stem height and tree ring growth (RW, EWW, LWW, and LWP), wood density (RD, EWD, and LWD), and stem form (ST, BT, and *d*_*i*_/DBH), following the three-step model selection approach (Zuur et al., 2009). First, we selected the random structure in the presence of the fixed structure (main effects and second-order interactions), and then, the optimal structure of the random component was determined based on restricted maximum likelihood (REML). Second, after selecting the random structure, model parameterization of the fixed component was fitted by maximum likelihood (ML). Relative stand density and stem height were entered as categorical and numeric variables in the fixed component in the models, respectively. Stand age and site quality were included in the random component, as they had no change in self-thinning trajectory (Bi et al., 2000; Sun et al., 2011). When necessary, the variance structure or autocorrelation was also modeled. The inference was performed after refitting the best models *via* REML.

The full models that included all of these independent variables took the form of the following equation:


(1) $${\text{Y}}_{i\,j}=\alpha_{0}+\alpha_{1}\times S_{i}+\alpha_{2}\times H_{j}+\alpha_{3}\times {\left(S\times H\right)}_{i\,j}+\beta_{k}+\delta_{t}+\varepsilon_{t\,i\,j\,k}$$

In model (1), the dependent variable Y*_*ij*_* is the value of the tree’s ring growth, wood density, and stem form for the *i*-th relative stand density with the *j*-th total tree height. α_0_, α_1_, α_2_, and α_3_ were estimated from the observed data. The fixed components included *S*_*i*_ and *H*_*j*_, and their interaction *S* × *H.* Potential differences in site quality and stand age were addressed by a random effect β*_*k*_* and δ*_*t*_*, respectively. The error term ε*_*tijk*_* contained the remaining unexplained variation for tree ring growth, wood density, and stem form on the *t*-th stand age from the *i*-th relative stand density with the *j*-th total tree height position at the *t*-th stand age from *k*-th site quality plots and is assumed to be independent and normally distributed with mean 0 and variance σ^2^.

ANOVA and pairwise Pearson correlation analyses were carried out between all of the variables to determine the significance and strength of their relationships among the different tree properties. All tests were performed at a 5% significance level and the statistical analyses were conducted using SAS software (SAS Institute Inc, 1999).

## Results

### Influence of Stand Density and Stem Height on Ring Growth

**FIGURE 2:**
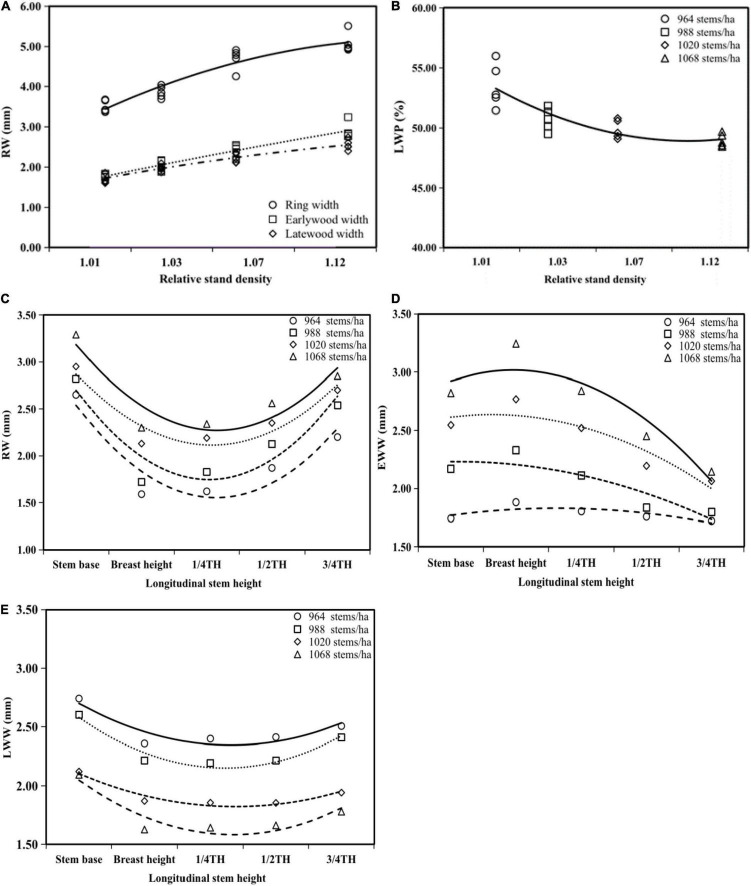
Variation of ring width (RW), earlywood width (EWW), latewood width (LWW), and latewood proportion (LWP) in the radial and longitudinal stem directions during self-thinning trajectory of the even-aged pure *S. tzumu* stands. **(A)** Influence of stand density on RW. **(B)** Influence of stand density on LWP. **(C)** Influence of stem height on RW. **(D)** Influence of stem height on EWW. **(E)** Influence of stem height on LWW.

Relative stand density significantly and strongly affected RW, EWW, LWW, and LWP (Table 2 and Figures 2A,B). There was a significant reduction in the average radial ring growth values of RW (−30.95%), EWW (−38.36%), LWW (−32.62%), as relative stand density decreased during the self-thinning of the *S. tzumu* plots. Relative stand density had an inverse effect on LWP, which increased by 7.2% with decreasing living stems per hectare (Table 3).

**TABLE 2:** Influence of the main and interactive effects of relative stand density and stem height on three dimensions, architecture, and wood properties.

Wood quality and stem form	Relative stand density (S)		Stem height (H)		S × H	
	*F*-value	Pr > *F*	*F*-value	Pr > *F*	*F*-value	Pr > *F*
**Ring growth**						
RW	50.377	0.0001	10.855	0.0256	0.631	0.217
EWW	13.896	0.0008	4.379	0.0271	1.349	0.145
LWW	47.753	0.0021	8.248	0.0183	0.434	0.359
LWP	1.647	0.0230	2.225	0.0296	0.547	0.468
**Wood density**						
RD	4.097	0.028	4.316	0.0474	1.600	0.861
EWD	5.732	0.049	3.739	0.0433	0.943	0.575
LWD	6.155	0.017	2.994	0.0415	1.296	0.434
**Stem form**						
ST	290.921	0.0053	2.35	0.192	1.320	0.951
BT	63.827	0.0085	1.852	0.273	0.349	0.339
*di*/DBH	0.051	0.4551	977.710	0.004	0.782	0.082

**TABLE 3:** Means of tree dimensions and wood properties in relation to relative stand density and stem height in the even-aged pure *S. tzumu* stands.

Stand density/stem height		Radial growth				Wood density			Stem form		
		RW (mm)	EWW (mm)	LWW (mm)	LWP (%)	RD (kg/m^3^)	EWD (kg/m^3^)	LWD (kg/m^3^)	ST	BT	*di*/DBH
Relative stand density	1.12	5.07*^a^*	2.90*^a^*	2.17*^b^*	49.88*^c^*	165.60*^d^*	155.86*^c^*	836.07*^d^*	0.83*^c^*	1.82*^c^*	101.08*^a^*
	1.07	4.69*^b^*	2.44*^b^*	2.24*^a^*	50.08*^bc^*	326.21*^c^*	192.99*^bc^*	1,074.61*^c^*	0.97*^c^*	1.47*^d^*	112.98*^a^*
	1.03	3.86*^c^*	2.02*^c^*	1.84*^c^*	50.71*^b^*	506.08*^b^*	224.94*^b^*	1,321.06*^b^*	1.29*^b^*	4.04*^a^*	55.35*^c^*
	1.01	3.51*^d^*	1.79*^d^*	1.72*^c^*	53.49*^a^*	708.58*^a^*	243.80*^a^*	1,591.17*^a^*	1.65*^a^*	3.43*^b^*	80.52*^b^*
Stem height (m)	Stem base	5.20*^a^*	2.32*^b^*	2.39*^a^*	82.83*^e^*	1,108.10*^c^*	523.67*^b^*	1,994.46*^c^*			
	Breast height	4.15*^d^*	2.56*^a^*	2.03*^c^*	95.20*^b^*	1,246.41*^a^*	563.00*^a^*	2,172.43*^a^*			
	1/4 TH	4.27*^cd^*	2.33*^b^*	2.02*^c^*	97.38*^a^*	1,214.14*^ab^*	485.42*^c^*	2,091.01*^b^*			
	1/2 TH	4.45*^c^*	2.06*^bc^*	2.04*^c^*	89.80*^c^*	1,188.62*^b^*	352.32*^d^*	1,912.46*^c^*			
	3/4 TH	4.95*^b^*	1.93*^c^*	2.16*^b^*	85.80*^d^*	1,042.13*^c^*	208.68*^e^*	1,699.39*^d^*			

*The letters (a–d) in the same column represent statistical comparisons (5% of significance), while different letters indicate significant differences between the value.*

Ring width, EWW, and LWW changed significantly from the stem base toward the stem apex (Table 2). There was a sharp decrease in RW from the stem base to breast height, and then a slight increase from breast height to 1/2 TH, and, finally, a rapid increase from 1/2 TH to 3/4 TH (Figure 2C). EWW exhibited a consistent pattern among relative stand density with a decrease between the stem base and 1/2 TH followed by a slightly increasing trend up to 3/4 TH (Figure 2D). The trend in LWW increased from the stem base to 1/2 TH and then decreased with an increase in the stem height (Figure 2E). Examination of the *F*-values showed that the influence of stem height on these variables was considerably lower than that of relative stand density and cambial age (Table 2).

**FIGURE 3:**
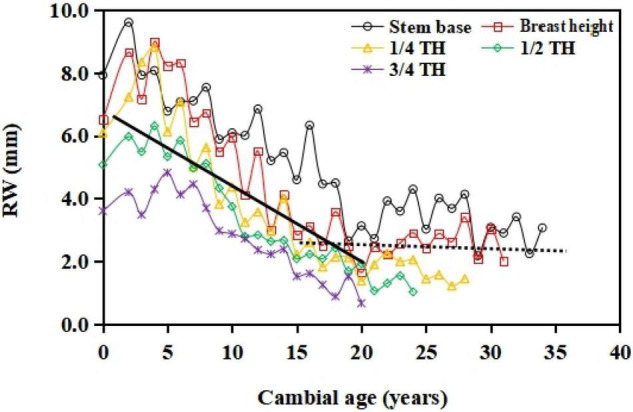
Within-tree trends in ring width (RW) during the self-thinning of *S. tzumu* stands. The different curves correspond to different stem heights. The crossing point between the black straight solid line and the dotted line shows the transition from juvenile and mature wood at the cambial age from the pith to the bark.

Along with the tree ring growth from the pith to the bark, RW showed a tendency to increase rapidly during the early growth stage and then subsequently decrease (Figure 3). The cambial ages with the largest RW values varied with the position along the longitudinal stem direction. The cambial age with the largest RW was 1.6 years at the stem base, 2.6 years at breast height, 3.0 years at 1/4 TH, 5.0 years at 1/2 TH, and 7.0 years at 3/4 TH. For the same cambial age, the width of the thickest ring decreased from the stem base to the apex. For example, RW values up to a cambial age of 7 years were 4.98, 4.22, 4.20, 3.76, and 3.55 mm/year at the stem base, breast height, 1/4 TH, 1/2 TH, and 3/4 TH, respectively. All of the individuals of different stand ages during the self-thinning trajectory showed similar axial variations. The discrimination between the juvenile wood and the mature wood was based on the cambial age in the cross-section disks. The intersection point between the juvenile/mature wood growth trends showed a cambial age of 16.8 years; thus, the juvenile wood was produced during the first 17 years. It was characterized by a rapid decrease in juvenile tree-ring width, whereas tree-ring growth with the mature wood almost remained constant after the following growth stage.

### Influence of Stand Density and Stem Height on Wood Density

**FIGURE 4:**
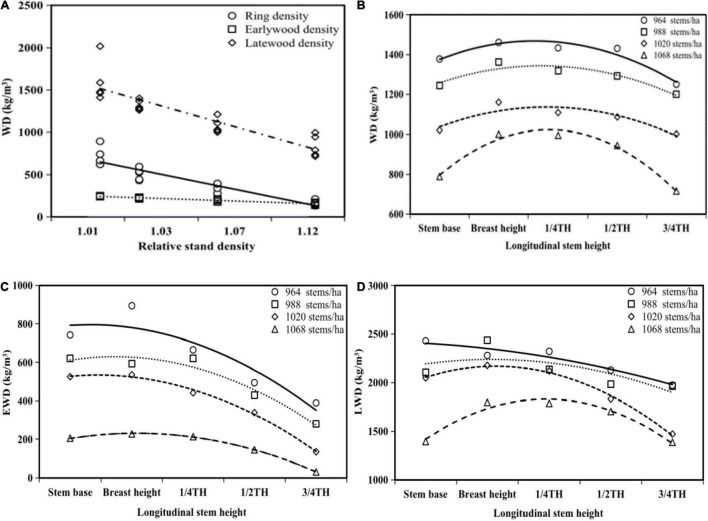
Variation of wood density (WD) **(A,B)**, earlywood density (EWD) **(C)**, and latewood density (LWD) **(D)** along the radial and longitudinal stem directions.

Relative stand density significantly influenced RD, EWD, and LWD (Table 2). The RD and LWD increased rapidly with decreasing relative stand density during the self-thinning phase. However, EWD increased slightly with decreasing relative stand density (Figure 4A). WD within a disk exhibited a sharp increase from the stem base to breast height, decreased gradually from breast height to 1/2 TH, and, finally, decreased rapidly from 1/2 TH to 3/4 TH (Figure 4B). EWD and LWD exhibited similar trends, increasing from the stem base to breast height, and then decreasing from breast height to 3/4 TH (Figures 4C,D).

### Influence of Stand Density and Stem Height on Stem Form

Relative stand density significantly and strongly influenced ST and BT, but it did not have any significant effect on *d*_*i*_/DBH (Table 2). A decrease in relative stand density resulted in decreased ST (Figure 6A), while BT and *d*_*i*_/DBH increased (Figures 6B,C).

**FIGURE 5:**
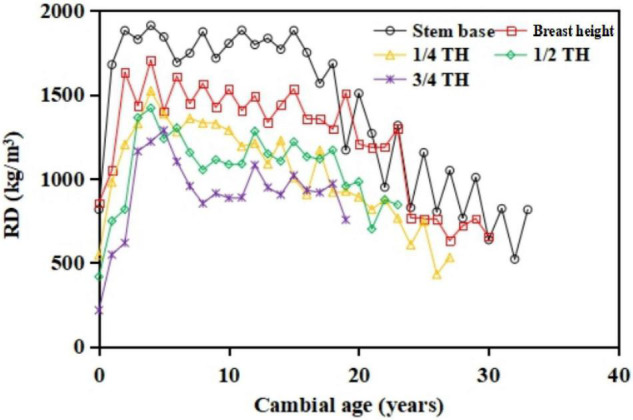
Within-tree variation of ring density (RD) during the self-thinning of the even-aged pure *S. tzumu* stands. The different curves correspond to the different stem height direction.

Regarding the radial variation of RD and LWD along with the radial disc, RD increased from the pith outwards until the maximum value was reached around 3–7 years, and then decreased with increasing cambial age in mature wood (Figure 5).

**FIGURE 6:**
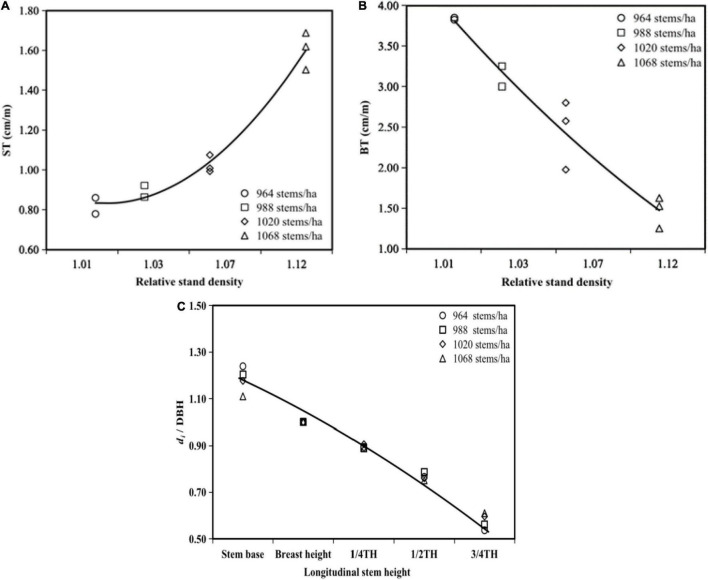
Variation of ST **(A)**, BT **(B)**, and *d*_*i*_/DBH **(C)** during self-thinning *S. tzumu* stands.

### Bivariate Correlations Among Ring Width, Wood Density, and Stem Taper

Ring width was positively correlated with EWW, LWW, and ST, and it was negatively correlated with RD, EWD, LWD, and BT. There was a significantly negative correlation between EWW and LWW. RD exhibited highly significant, positive correlations with EWD and LWD, and LWD was negatively correlated with *d*_*i*_/DBH (Table 4).

**TABLE 4:** Pearson’s correlation coefficients (and significances at the 5% level) between RW, WD, and ST during self-thinning *S. tzumu* stands.

Ring features and stem form	RW	EWW	LWW	RD	EWD	LWD	ST	BT
EWW	0.948**							
LWW	0.898**	−0.729**						
RD	−0.725**	−0.110*^ns^*	−0.092*^ns^*					
EWD	−0.529*	0.123*^ns^*	−0.405*^ns^*	0.960**				
LWD	−0.417*	0.079*^ns^*	−0.425*^ns^*	0.581**	−0.510**			
ST	0.705**	0.247*^ns^*	−0.063*^ns^*	0.219*^ns^*	0.178*^ns^*	0.272*^ns^*		
BT	-0.847**	−0.004*^ns^*	−0.026*^ns^*	−0.498*^ns^*	−0.596*	0.027*^ns^*	0.275*^ns^*	
*di*/DBH	−0.383*^ns^*	−0.433*^ns^*	−0.193*^ns^*	−0.180*^ns^*	−0.139*^ns^*	-0.573*	−0.104*^ns^*	−0.112*^ns^*

ns, non-significant at P = 0.05;*, significant at P = 0.05; **, significant at P = 0.01.

## Discussion

This study showed the patterns of tree ring growth, wood density, and stem form along a stand self-thinning trajectory. Our findings indicated that, as the *S. tzumu* plots density decreased, there were improvements in wood quality and tree form. We found a strong negative correlation between ring growth and wood density of the even-aged pure *S. tzumu* stands during the self-thinning phase. It is worth mentioning that these findings contrast with several other studies on conifer, ring-porous hardwood, or diffuse-porous hardwood species (Bergès et al., 2000; Genet et al., 2012; Clough et al., 2017). For example, softwood pine species show a parallel trend in tree ring width and tree RD (Vannoppen et al., 2018). A positive correlation between tree ring width and tree ring wood density exists in ring-porous species like oak or ash (Zhang et al., 1993). In diffuse-porous hardwood species like spruce, beech, and poplar, tree ring wood density is almost independent of tree ring width (Franceschini et al., 2010; Diaconu et al., 2016).

For *S. tzumu* plantations, the relative stand density was positively correlated with ring width and negatively correlated with tree ring wood density. In this way, with the decrease in relative stand density because of competition-induced mortality, there was a decrease in ring width and an improvement in wood density due to greater mature wood formation initiating approximately 17 years after planting. This contrasts with other studies in which relative stand density was positively correlated with tree ring wood density and negatively correlated with tree ring width (Pritzkow et al., 2014; Zeller et al., 2017; Newton, 2019), which may partly be due to intra-specific growth variability, climatic conditions, site characteristics, and planting density (Schuldt et al., 2016; Zeller et al., 2017; Islam et al., 2019).

As the cambium cells become mature, the tendency is to reduce the width of the growth ring and to produce mature wood, which is characterized by a higher LWP compared with juvenile wood (Li et al., 1998; Zhu et al., 2000; Alteyrac et al., 2006). This has been observed for *Larix kaempferi*, such that high growth rates resulted in lower-density wood during the juvenile period (Zhu et al., 2000). With the increase in ring width during the early years of tree growth, the latewood percentage decreases significantly in juvenile wood. However, in the mature wood, there is a decrease in ring width and, consequently, an increase in the latewood percentage probably confounded with stand age effects (Alteyrac et al., 2006).

Part of the effect of stand density is probably also the effect of stand age. As the cambium cells become mature, there is a trend of decreasing ring width and increasing LWP, leading to mature wood formation (Zhu et al., 2000; Alteyrac et al., 2006; Darmawan et al., 2013). For *S. tzumu*, the juvenile wood was produced during the first 17 years. This indicates that when relative stand density exceeded 1.07, between the ages of 10 and 20 years, there was a predominance of juvenile wood being formed. On the contrary, as the relative stand densities declined, which occurred at the ages of 30 and 40 years, there was a predominance of mature wood formation that is characterized by an increase in LWP, which is of a higher density. For *Picea mariana*, the simultaneous variation of juvenile wood diameter and tree diameter along the stem direction leads to a constant proportion of juvenile wood (Alteyrac et al., 2006). These authors indicate that the proportion in volume of the juvenile wood remains constant along the longitudinal stem direction. Also, the authors found that juvenile wood increased with relative stand density. The decrease in ring width and increase in LWP support the hypothesis of an increase in the proportion of mature wood with increasing cambial age of *S. tzumu* during the self-thinning phase. The results indicate that there was an increase in the diameter of the tree and the formation of mature wood with significant improvements in the quality of *S. tzumu* wood during the self-thinning phase as RD increases, especially after the age of 17 years.

Competition was maintained at high levels during the self-thinning phase, and RD decreased along the axial stem direction from breast height to the stem apex. This was related to increasing EWW and decreasing LWW along the stem from the base to the apex. This trend in RD along the stem probably results from the higher proportion of juvenile wood at the stem apex compared with that at lower stem positions along the stem (Mäkinen and Isomäki, 2004).

The effects of stem height were often significant on stem dimension and stem form. The axial variation at a specific stem height proceeds downward and upward along a stem as a stand undergoes self-thinning (Pretzsch et al., 2018), which explains the somewhat larger dimensions and wood properties at the tree level. In our study, EWW increased from 1.30 m TH to the stem apex, and it also increased rapidly from breast height to the stem base. Accordingly, narrower tree ring widths at the highest and at the lowest parts of the stem decreased the tree ring wood density and stem taper during the stand self-thinning phase. When the restrained tree death causes gaps, the change of internal resource distribution along the surviving stem may be more inclined to diameter increase and crown expansion (Castle et al., 2018). *S. tzumu* is a shade-intolerant deep-rooting tree species, and the rapid growth of *S. tzumu* individuals can enhance its access to light and outcompete the slow growth of neighboring trees.

In summary, as relative stand density decreased throughout the self-thinning phase, wood density and LWP increased, whereas growth ring width and stem taper decreased. From a silvicultural perspective, we showed that, in conditions similar to those in our study, one can expect the predominant formation of mature wood to start at about 17 years after planting. Our analysis of wood properties shows the importance of considering the position along the stem when examining wood properties. Such information is valuable for the improvement of the silviculture and wood utilization of *S. tzumu* plantations.

## Conclusion

During the self-thinning of the even-aged pure *S. tzumu* stands, ring growth values of RW, EWW, and LWW decreased, but LWP increased; RD, EWD, and LWD increased; ST decreased, while BT and *d*_*i*_/DBH increased. Decreased tree growth during self-thinning trajectory was offset by improved wood density and stem form. Tree ring growth closer to the apex of the stem and the lower parts of the stem decreased the tree ring wood density during stand self-thinning trajectory. The shift in the internal resource allocation along the stem might prefer to maximize end-product value of timber.

This study highlights the important influence of stand density on tree ring growth, wood density, and stem form. A key finding was that the stem height did not significantly interact with stand density for any of the key wood properties during the self-thinning phase, which suggests the additive effects of stand density and stem height have on wood properties.

## Data Availability Statement

The raw/processed data required to reproduce these findings cannot be shared at this time as the data also forms part of an ongoing study. Further inquiries can be directed to the corresponding author.

## Author Contributions

HS and JJ conceived and designed the experiment. SD performed the experiment and collected the data. HS analyzed the data and wrote the initial draft of the manuscript. AS, TP, and DF edited and revised the final version of the manuscript. All authors approved the final version for publication and agreed to be held accountable for the content therein.

## Conflict of Interest

The authors declare that the research was conducted in the absence of any commercial or financial relationships that could be construed as a potential conflict of interest.

## Publisher’s Note

All claims expressed in this article are solely those of the authors and do not necessarily represent those of their affiliated organizations, or those of the publisher, the editors and the reviewers. Any product that may be evaluated in this article, or claim that may be made by its manufacturer, is not guaranteed or endorsed by the publisher.
